# First-passage time analysis of a one-dimensional diffusion-reaction model: application to protein transport along DNA

**DOI:** 10.1186/1471-2105-12-S10-S18

**Published:** 2011-10-18

**Authors:** Michael L Mayo, Edward J Perkins, Preetam Ghosh

**Affiliations:** 1 Environmental Laboratory, US Army Engineer Research and Development Center, Vicksburg, MS 39180, USA; 2 Department of Computer Science, Virginia Commonwealth University, Richmond, VA 23284, USA

## Abstract

**Background:**

Proteins search along the DNA for targets, such as transcription initiation sequences, according to one-dimensional diffusion, which is interrupted by micro- and macro-hopping events and intersegmental transfers that occur under close packing conditions.

**Results:**

A one-dimensional diffusion-reaction model in the form of difference-differential equations is proposed to analyze the nonequilibrium protein sliding kinetics along a segment of bacterial DNA. A renormalization approach is used to derive an expression for the mean first-passage time to arrive at sites downstream of the origin from the occupation probabilities given by the individual transport equations. Monte Carlo simulations are employed to assess the validity of the proposed approach, and all results are interpreted within the context of bacterial transcription.

**Conclusions:**

Mean first-passage times decrease with increasing reaction rates, indicating that, on average, surviving proteins more rapidly locate downstream targets than their reaction-free counterparts, but at the price of increasing rarity. Two qualitatively different screening regimes are identified according to whether the search process operates under “small” or “large” values for the dissociation rate of the protein-DNA complex. Lower bounds are placed on the overall search time for varying reactive conditions. Good agreement with experimental estimates requires the reaction rate reside near the transition between both screening regimes, suggesting that biology balances a need for rapid searches against maximum exploration during each round of the sliding phase.

## Background

Information in the form of nucleotide sequences is processed into RNA during transcription, which are then processed into proteins responsible for regulating signaling pathways and other elements of cellular chemistry. Proteins, called transcription factors, are transported to the DNA by diffusion, wherein they attach to DNA “receptors,” forming semi-stable complexes. Initiation of transcription occurs after a search along DNA for specific terminating sequences; their presence attenuates the affinity of RNA polymerase to the promoter region of the targeted gene, increasing or decreasing transcriptional activity. Early experimental work on the *lac* repressor in *E. coli*[1,2], and the seminal theoretical works of Winter, Berg, and von Hippel [3-5], laid the groundwork in the current understanding of protein-search kinetics. Targets (i.e., transcription initiation sequences) are located after conducting a one-dimensional search along the DNA, transported there by diffusion through the cytoplasm, wherein sliding is interrupted by micro- and macro-hopping events along single segments and intersegmental protein transfers across DNA strands in close proximity under close packing conditions. Such rounds of *jumping* and *sliding* across the DNA are now collectively referred to as “facilitated target location” [6]. In vivo, pure sliding is complicated by protein-phosphate backbone interactions [7], energetic and steric restrictions (e.g. DNA conformational changes [8,9]), access limitations induced by diffusion gradients [10], macromolecular crowding [11], as well as proteins binding to multiple segments [12], to name just a few. Moreover, single molecule experiments conclude that protein association to the DNA dominates dissociation, wherein the resulting protein-DNA complex is relatively stable during the sliding process [13-15].

Here, we investigate the implications for this stability of the protein-DNA complex on the overall search process by formulating the protein-transport problem as a diffusion-reaction process in one dimension; the speed of the overall search is quantified in terms of the total time, *t_s_*, needed to find the transcription initiation sequence, which is the sum of the times spent sliding and jumping during each round:(1)

In this expression, *t_i_* (Λ) is the time needed for the protein to traverse a length Λ (in units of base-pairs, bps) for each sliding event, *t*_3*D*,*i*_ is the time a protein spends free of the DNA beteween each round of sliding, and *N_s_* is the number of sliding rounds in the search. Equation 1 may be simplified by taking its average, , across all rounds of sliding and jumping [16], resulting in:(2)

wherein  labels the average search time; 〈*t_i_* (Λ)〉 = *t*_Λ_ labels the average time needed to explore a length Λ from the site of association, termed the exploration length [17]; 〈*t*_3*D*,*i*_〉 = *t*_3*D*_ labels the average time a protein spends free of the DNA during the search. Furthermore, it has been argued that biology is optimized to minimize the overall search time [16], so that *t*_Λ_ = *t*_3*D*_. Under this assumption, Eqn. 2 may be expressed as:(3)

So, under suitable assumptions, the calculation of the total search time is reduced to an estimation of the length of the sequence actually searched, *L*, the exploration length, Λ, and the average time spent sliding during each round, *t*_Λ_.

While others have investigated the transport properties of the protein-DNA complex in the presence of reversible binding kinetics [3], our analysis of the overall search times, based on Eq. 3, reveals new insight into the extraordinary speed of the target-location process. We frame our discussion in terms of a first-passage time analysis, from which we estimate *t*_λ_ for a one-dimensional diffusion-reaction model proposed below, and use it to show that the reaction rate serves to screen proteins from downstream exploration of the DNA-a fact that has implications for the timely response of mRNA transcription to the presence of transcription factors.

## Methods

Proteins are much smaller in length than the DNA they attach to. Indeed, ignoring the conformation of either protein or DNA, an *E. coli* genome is ~ 10^5^ times longer than a protein bound to it. A typical genome is ~ 10^6^ bases (~ 1*/*3 mm, given that the distance between bases is Δ*l* = 1/3 nm), and the median “length” of an E. coli protein is approximately 278 amino acids [18], or ≈ 278*Å*; the ratio of protein to genome length is therefore approximately 8 × 10^–5^. So, the DNA appears appears to be a long segment of nucleotide bases, as viewed from the perspective of DNA-bound proteins. It is natural, then, to model a long sequence as a one-dimensional lattice, wherein the lattice sites correspond to the base-pair positions of the underlying DNA.

**Figure 1:**
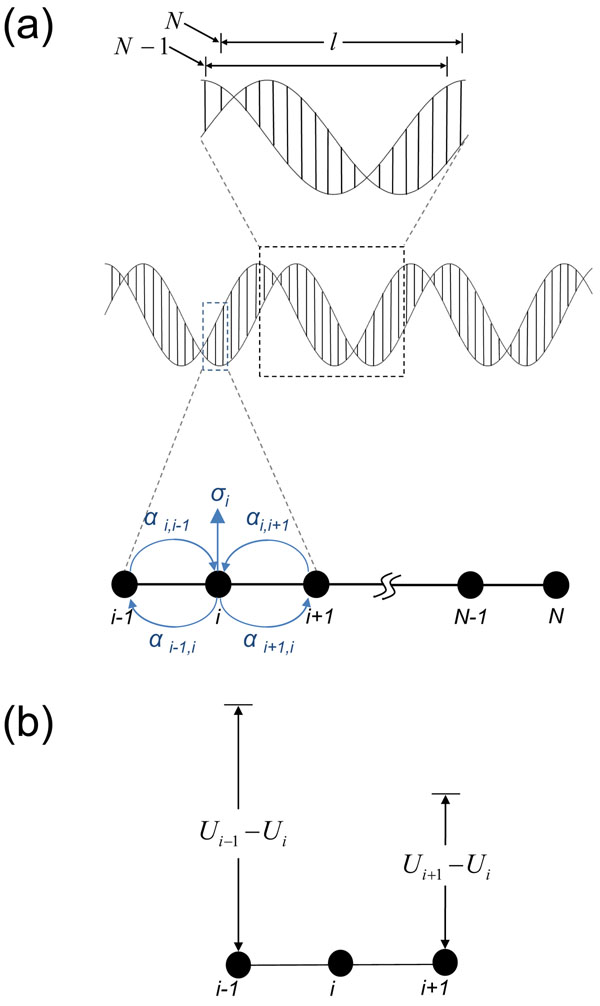
**Diffusion-reaction model of protein transport along DNA** (a) Nucleotide base-pairs correspond to the sites of a model one-dimensional lattice; (b) transport of proteins to adjacent bases is occurs according to the difference in the binding free energies of the adjacent sites with the current one.

For the present study, sliding of the protein occurs by diffusion across a segment of DNA, wherein this segment is modeled by a one-dimensional lattice (Fig. 1). Diffusion begins from site *i* = 1, termed the origin, and terminates upon successful dissociation of the protein-DNA complex at any intermediate site *i* = 2,3,…, *N –* 1, wherein *N* is the number of bases in the modeled segment. The exact biological terminating mechanism of the target-location event is ignored: we merely assume that a particle trap is hosted at site *i* = *N*, which is implemented as a perfectly absorbing boundary condition (see below). The exact way in which the Hydrogen donor and acceptor bond-patterns destabilize the minimum interaction potential at the terminating site established during the protein-slide [19], is of no direct importance to the problem at hand.

### Energetic basis for protein diffusion along DNA

While a protein is sliding, it is subject to forces originating from interactions with local sections of the DNA (e.g. nucleotides), the surrounding medium (e.g. cytoplasm), and other nonsequence interactions (e.g. the phosphate backbone). The net interaction between protein and DNA has a component that depends strongly on the actual nucleotide sequence, by which the generally positively charged proteins interact with the generally negatively charged bases. Following tradition [16,20,21], the free energy related to the binding of a protein with the DNA at site *i* (with respect to the cytoplasm), *U_i_*, is decomposed into two contributions: the specific, or sequence-dependent, component, , and the nonspecific, or sequence-independent, component, *E_ns_*. This decomposition gives:(4)

wherein  is a binding sequence (*s_i_* ∈ {*A*,*C*,*G*,*T*}) of length *l*.

In a standard approximation, the contribution of each base to the sequence-specific binding energy is considered to be additive [20-22], and is inferred using a weight matrix method applied to binding sequence profiles. This has been carried out for the bacterial transcription factors purE (E. coli), fruR (E. coli), and Hl1635 (H. inf.) [16], wherein the binding free energies associated with sequences of the entire genome were found, to good approximation, to be Gaussian-distributed for sufficiently long sequences. On the basis of this evidence, we consider the binding free energies of a generic bacterial protein sliding along the genome to be drawn from the following probability distribution [16]:(5)

wherein *μ* is the standard deviation of the distribution.

Although varying correlation lengths in the protein-DNA binding free energy may arise in several ways-such as directly, from the nonspecific energy [23], DNA curvature [8], and nonrandom sequences with high protein affinity (e.g. AT/GC-rich isochors [8]), or effectively, by mapping correlated micro-hops to a sliding process by “reducing” the free energy barriers to transport [9,24]-on larger scales, Eqn. 5 provides evidence to support an assumption of an uncorrelated binding free energy landscape. This idea has formed the basis of other studies [16].

Such an energetic landscape is quantified by its roughness, *μ*, which measures the value of an average fluctuation:(6)

and estimated previously to be *μ* ~ *k_B_T* under biologically relevant conditions (written in terms of the system temperature, *T*, and Boltzmann’s constant *k_B_*) [8,16].

Transport from one site to either adjacent one is inhibited by an energetic barrier conceptualized as the sides of a box with height *U*_*i*±1_ – *U_i_* (Fig. 1). It can be shown that the transition probability to reach adjacent sites is related to the uninhibited transition rate, *ν*, by an Arrhenius-type equation [16,25]:(7)

Equation 7 describes a DNA segment in thermal contact with cytoplasm of temperature *T*, 1/*β* = *k_B_T* is the absolute temperature, and the factor of one half results from normalization. These transition probabilities are, in general, not symmetric; i.e., *ω*_*i*+1,*i*_ ≠ *ω*_*i*,*i*+1_. Because the binding energies, *U_i_*, are considered here to be Gaussian-distributed random variables, equation 7 implies that *ω*_*i*+1,*i*_ + *ω*_*i*–1,*i*_ ≤ *ν;* the difference being the probability per unit time for the protein not to jump to either site, termed the sojourn probability [26]. Furthermore, at any time during the walk, the protein-DNA complex may dissociate with rate *r_i_* (Fig. 1), triggering extraneous protein macro- and micro-hopping events.

The biological problem of protein diffusion along the DNA under dissociative conditions is therefore mapped onto the generic problem of one-dimensional diffusion that proceeds according to randomly chosen transition probabilities, and subject to reactive conditions with the model lattice. In the next section we provide nonequilibrium transport equations that, collectively, serve as the mathematical foundation for the sliding process under study.

### Transport equations for the one-dimensional model

Given a protein that is currently associated to the lattice site *i*, it may move to adjacent sites according to the following “rules.” It may i) hop to site *i* + 1 with probability *α*_*i*+1,*i*_ = *ω*_*i*+1,*i*_/*ν*; ii) hop to lattice site *i –* 1 with probability *α_i_*_–1,_*_i_*; iii) react with, or dissociate from, the lattice site *i* with probability *σ_i_* (1 – *α*_*i*+1,*i*_ – *α*_*i*–1,*i*_), wherein *σ_i_* = *r_i_*/*ν* is the probability to react with the lattice at site *i* in time 1/*ν* . Dissociation from the DNA may occur, for example, by interacting with on-site obstacles during the slide [27].

For diffusion beginning from the origin, *i* = 1 and terminating at site *i* = *n*, the occupation probability for any site *i* along the segment, *p*_*i*,1_(*t*), at the elapsed time *t*, can be found by formulating the diffusion rules i-iii) above as a set of difference-differential equations for *n* lattice sites:(8)(9)(10)

Note that reaction rates, transition probabilities, and times, are scaled in units of 1/*ν*, rendering the transport equations dimensionless.

Using a somewhat different approach, Slutsky and Mirny [16] estimated *ν* ~ 10^8^*Hz–a* value which justifies the use of continuous-time differential equations for the protein diffusion problem. Additionally, the probability is sharp at the origin, so that initial data for equations 8-10 are given by:(11)

wherein *δ*_*i*1_=0 (when *i* ≠ 1) or 1 (when *i* = 1) is the Kronecker delta.

Note that boundary conditions 8 and 10 must be specified to provide a unique set of occupation probabilities for the entire lattice; we have chosen to provide a reflecting boundary condition at the source of the diffusion. Although the diffusion properties are initially influenced by this boundary condition, boundary effects vanish for the late times and distances that are relevant at biological time (~ 1 second) and length scales (~ 100 bases), because of the uncorrelated nature of the diffusive trajectory.

### Renormalization of the first-passage time distribution

The mean first-passage time, *τ_i_*, measures the average time for a diffusing object to arrive at a target site *i* for the first time, and is often used as an estimate for reaction rates of creation/descruction across a boundary, and transit times through a medium, among other phenomena of interest [28]. For a protein diffusing along the DNA, the mean first-passage time (MFPT) is the average *minimum* search time needed to locate a target site downstream from the site of association with the DNA, because transport is always terminated at the target sequence. As transcription factors at the promoter sequences of a gene either recruit or inhibit RNA polymerase binding, the rate at which they first arrive at the promoter sequence might also provide a rough estimate of a gene’s transcriptional activity.

The occupation probabilities of the difference-differential equations 8-10 can be written as [28,29]:(12)

Here, the term *p*_*i*,*i*_ (*t* – *t*′) denotes the probability of the protein transported to site *i*, to leave and return again by sliding to site *i* in time *t* – *t*′ ≥ 0; the protein may leave and return many times in this interval. Finally, the term *F*_*i*,1_ (*t*′) gives the probability per unit time that a protein will reach the site *i* for the first time, starting from the origin, at time *t*′ ≤ *t*.

For nonzero reaction rates, however, we typically find , which is a consequence of the loss of probability by dissociation of the protein-DNA complex. So, using this distribution to compute an average over all times is not physically meaningful. Instead, we note that the first-passage distribution for the terminal site, *i* = *n*, is equal to the rate in which probability enters into it:(13)

because any walker that enters is restricted from leaving to contribute to occupation probabilities of any other sites, according to Eq. 10.

As mentioned, not all walkers that begin from the origin will survive to be counted as crossing the terminal site *n*–only the properties of those proteins surviving to this site are of interest. Equation 13 should therefore be renormalized to account for only those walkers surviving the entire transit to site *n*:(14)

This process may be extended to every site along the lattice by placing the terminal trap at successive sites, and computing the first-passage distribution according to the procedure described above, for each one. So, for each site that is treated as a terminal one, *n* = 2, 3,…,*N*, we have:(15)

Note that the first-passage time for the origin is trivial, *τ*_1_ = 0.

### Monte Carlo simulations

Exact numerical simulations of the diffusion process, as defined above by the diffusion rules i-iii), were conducted by means of Monte Carlo simulation to study the diffusion-reaction process in terms of the mean first-passage time for a protein to reach a downstream target site. Individual trajectories were simulated, and the first-passage times were recorded for each site along the lattice. An arithmetic average of these first-passage times gives an approximation of the mean (equation 15).

**Figure 2:**
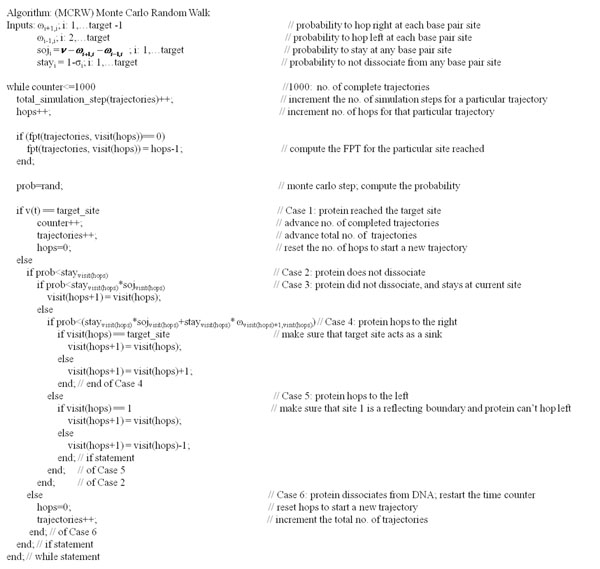
Pseudo code for the Monte Carlo Random Walk (MCRW) simulations of the diffusion-reaction process over a lattice of *N* sites

The simulation, termed Monte Carlo Random Walk (MCRW), begins when a random walker is placed at the origin of the lattice, and several checks are made according to the diffusion rules i-iii) during the transport process, and at each time-step (in units of 1/*ν*). Figure 2 illustrates the pseudo-code for MCRW, logically outlined as follows:

1. A 2D matrix, *total_simulation_step*, records the number of jumps experienced by the protein for each unique trajectory.

2. A 2D matrix, *fpt*, records the first-passage times for all the lattice sites visited for each trajectory.

3. A 1D array, *visit*, records the particular site that is visited for each jump in any particular trajectory. This array is overwritten upon termination of each trajectory, and can be used to compute other statistical metrics, such as the root-mean-square displacement, among others.

This simulation terminates when a random walker survives to reach the target site, for a total of 1000 times, wherein a counter for complete trajectories is incremented and another unique trajectory begins. Similarly, if a reaction event returns TRUE, the current trajectory terminates and another is started again at the origin, wherein reflecting boundary conditions (equation 8) are given.

Note that while we have only discussed the measurement of the mean first-passage times using the algorithm MCRW, it is straightforward to compute the root-mean-square displacements for the different trajectories, as well as other statistical endpoints as they vary with the dissociation probability, random energy background, target site, and so on.

## Results

### Preferential selection of fast-moving proteins under reactive conditions

**Figure 3:**
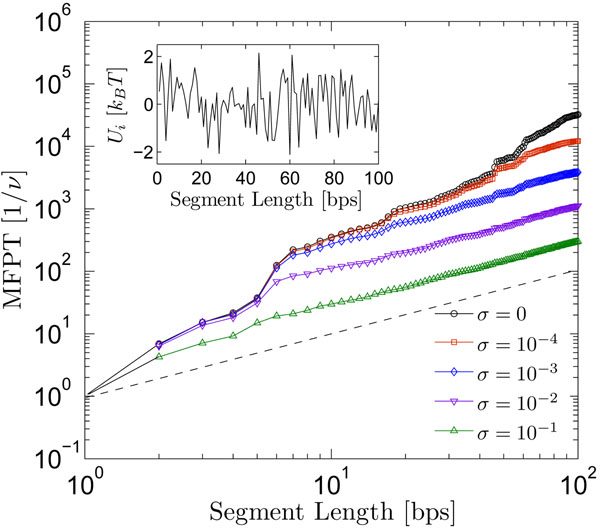
**Mean first-passage times for various reactive conditions.** Mean first-passage times decrease with increasing reactivity, as compared with the reaction-free path (*σ* = 0). The dashed line denotes the path in which every diffusion step is away from the origin. All values are computed using the binding free energy profile shown inset, characterized by roughness *μ* = 0.75*k_B_T*.

Figure 3 illustrates the mean first-passage times for a searching protein along the DNA in one particular realization of the free energy profile (*μ* = 0.75*k_B_T*), for a sequence of 100 bases in length, chosen because experiments on the BbvCI restriction enzyme suggest that Λ = 50 bases [30], but it is also long enough to minimize the effects of the reflecting boundary at the origin. It has been argued that the reaction rate depends almost entirely on nonspecific energy contributions to the total binding free energy [16], *E_ns_*, which is not dependent on the actual nucleotide sequence. We adopt this view by setting the reaction rates for each site equal to one another and dropping their indeces, *r* = *σν*. The influence of site-dependent reaction rates are considered below.

The mean first-passage time generally decreases with increasing reaction rate *σ*, demonstrating that only the “faster” trajectories-i.e., those clearing a maximum of bases in an equivalent amount of time-survive along the segment during each sliding phase. However, as *σ* increases, all trajectories become increasingly rare, in the sense that downstream sites are not visited as often as the ones associated with smaller *σ*. This indicates that transcription cannot entirely rely upon sliding as the main transport mechanism-an idea generally accepted since the early seminal works (see Background). If sliding were the only mechanism, a balance between minimizing search times while maximizing the length of individual slides might be required to most efficiently use the limited transcription factor proteins available in the cytoplasm (there may be as few as ~ 10-100 transcription factors present in the cytoplasm at any given time). However, since dissociation initiates the bulk diffusion involved in micro- and macro-hopping of the protein along the DNA, increasing distance covered and decreasing total search times, a larger reaction rate (but not too large) may actually support the search process by increasing the number of bulk excursions, allowing for the molecule to travel dominantly by 3D diffusion to distal DNA binding sites.

In the reactive cases, an increase in *σ* filters all but the superdiffusive trajectories (*x* ~ *t^d^*, *d* > 1/2) from those of the reaction-free case, leaving those that scale directly with time: *x* ~ *t*. This speed-up is consistent with intuition, in that ever faster trajectories should increasingly acquire the properties of the maximal one. This clearly cannot hold in the limit *σ* → 1, as no molecules survive on the lattice to be transported anywhere downstream of the origin.

**Figure 4:**
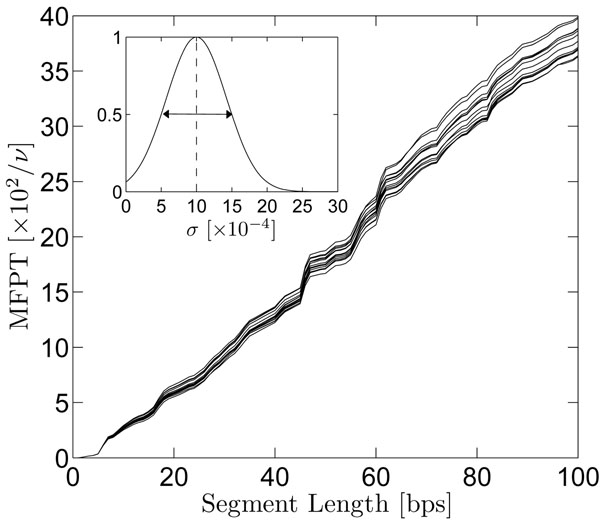
Sensitivity of the mean first-passage time with the reaction/dissociation rate

The sensitivity of the model to fluctuations in the reaction/dissociation rate are shown in Fig. 4. Reaction rates for each site, *σ_i_*, were chosen according to the distribution shown within the figure inset. Here, the full width at half maximum of the distribution is chosen to be equal to the mean 〈*σ_i_*〉 = 10^–3^. Even under such a broad distribution (or equally, conditions of large fluctuations), the mean first-passage times are remarkably stable across the length of the shown segment, supporting the use of a site-independent reaction rate used throughout this article.

### Larger energetic fluctuations slow a purely sliding target search

**Figure 5:**
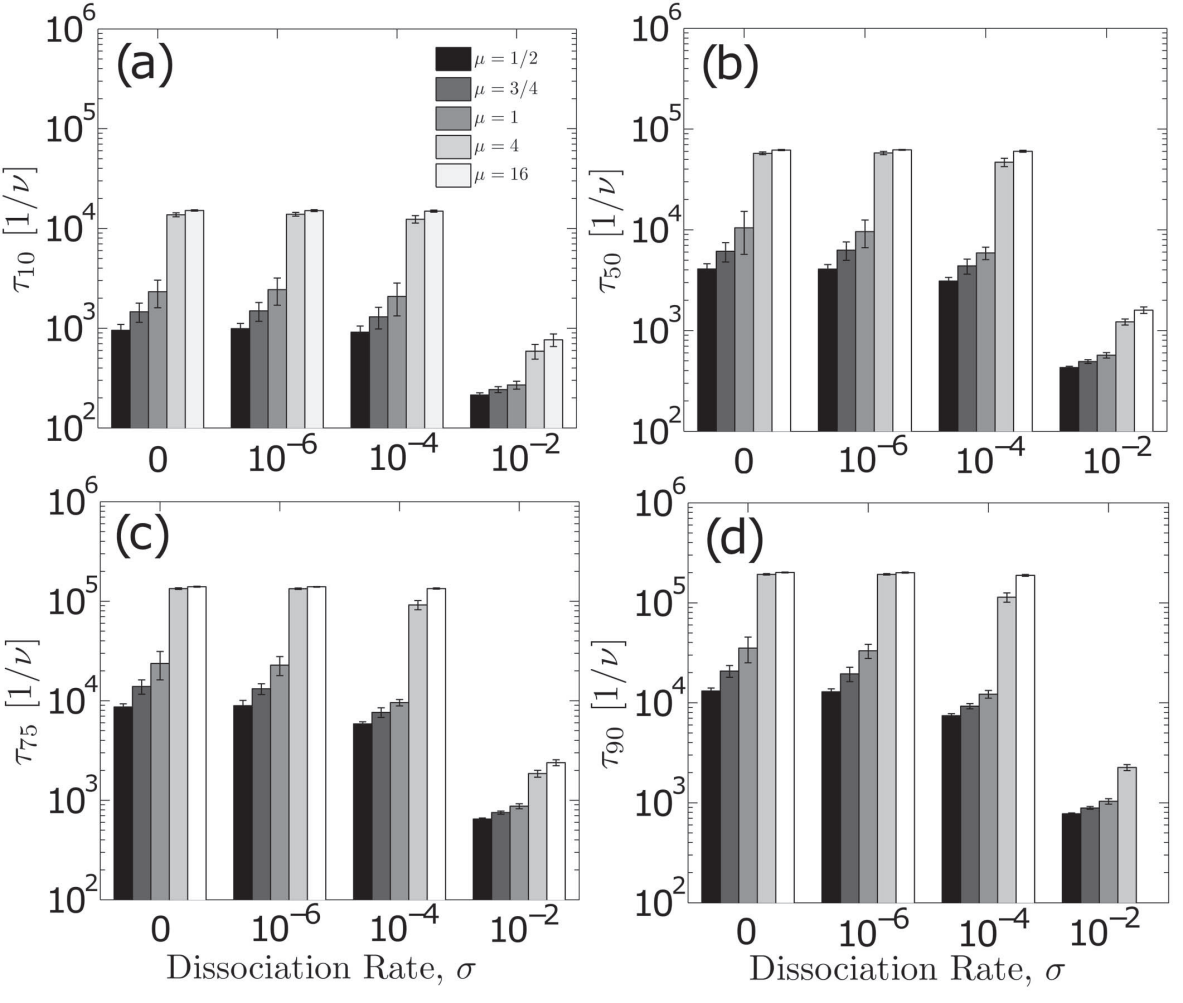
**Roughness dependence of the disorder-averaged mean first-passage times for various locations along a segment of DNA.** Mean first-passage times (y-axis) reflect the arithmetic average of 20 realizations of the free energy profile for the conditions shown. Roughness values are expressed in units of *k_B_T*. Panels (a) through (d) characterize the influence of differing roughnesses at varying positions along the segment.

Fluctuations in the free energy profile are quantified by the roughness, *μ*, equation 6. Figure 5 illustrates the disorder-averaged mean first-passage times (y-axis) for several locations along the segment as a function of the parameters *μ* and *σ*. The first-passage time characteristics are roughly the same for all lattice sites: smaller energetic fluctuations allow for the fastest transits under most reaction conditions. As the distribution, equation 5, becomes sharper, the energetic barriers to transport vanish, and the probability for an adjacent transition tends to 1, i.e. *α*_*i*+1,*i*_ + *α*_*i*–1,*i*_ → 1.

Interestingly, roughness values ranging from *μ* = 4*k_B_T* to 16*k_B_T* provide very similiar transport rates. As the distribution, equation 5, becomes wider, larger energetic barriers quench the transition frequency to adjacent sites, increasing residence times at the current site and slowing the sliding component of the overall search. Other studies suggest that a purely energetic terminating process would require *μ* ≥ 4*k_B_T*[16], which, according to figure 5, generates slower searches under all reactive conditions. The “optimal” roughness reported in these studies, *μ* = 0.75*k_B_T*, resides within a region of low variation in the mean first-passage times across a wide range of energetic conditions, wherein the roughness spans values ranging from *μ* = 1/2 to 1. Distrubtions adopting such values generate similar search times; such homogeneity under changing conditions provides a large degree of flexibility in the search process to many different types of proteins, and may provide tolerance against disruptions, such as temperature fluctuations.

### Survival times of proteins associated to DNA

Because the protein-DNA complex dissociates, exploring proteins are increasingly rare the further away from the origin they travel. The survival probability, Σ (*t*), quantifies this qualitative feature by giving the probability to find a protein anywhere along the segment as a function of time. Often of interest is the half-life of the protein-DNA comlex, *T*_1/2_, which is the time required for the survival probability to be exactly 1*/*2, and is found by inverting the following expression:(16)

**Figure 6:**
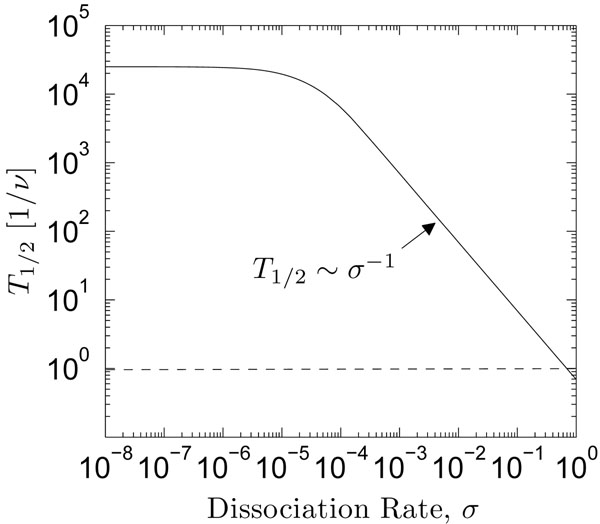
**Dependence of the half-life on the reaction rate.** Times (y-axis) mark the event at which the probability to find a protein anywhere along the model lattice is exactly 1/2, depending on the reaction/dissociation rate *σ* (x-axis).

Equation 16 is computed using the model energy profile inset of figure 3 (*μ* = 0.75*k_B_T*), and the results are illustrated within Fig. 6 This figure suggests that for any downstream exploration to occur by sliding alone, the dissociation rate of the protein-DNA complex must be very small, because only the fastest molecules survive to significantly explore downstream sites for increasing reaction rates. Moreover, the power-law type scaling behavior, *T*_1/2_ ~ *σ^γ^*, transitions from *γ* = 0 to *γ* = –1 rather quickly at approximately *σ* = 10^–4^, indicating that the influence of proteins trapped by the far boundary of the segment (*i* = *N*) washes out the contributions from any reactions with the medium for smaller *σ*. However, dissociation events dominate the half-life behavior for larger values of *σ*.

### Validation of the model and its biological implications

As described in the Background, equation 3 provides an expression for the optimal overall time for a search, and depends on the estimation of two quantities: *L* and *t*_λ_, given Λ = 50 bases. If we assume the span of the sequence that is actually searched is on the order of the length of the genome, *L* = 10^6^ bases, and we make the assumption that the search process terminates the first time a target sequence is found, *t*_λ_ = *τ*_50_, then Eq. 3 reduces to:(17)

**Figure 7:**
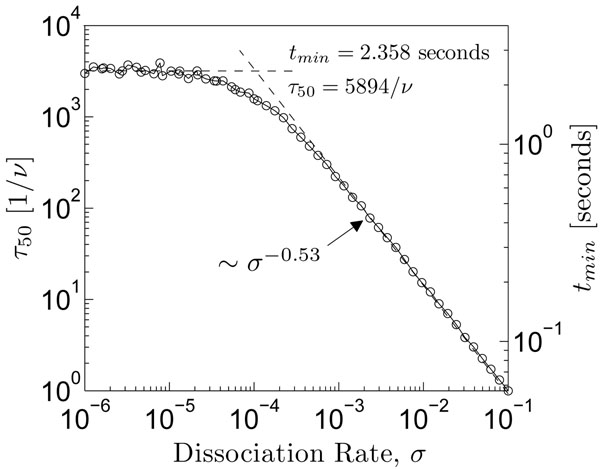
**Predictions for minimum target-location times**. Mean first-passage times required to traverse 50 bases are shown on the left axis. Minimum search times, as predicted by the diffusion-reaction model, are expressed on the right axis.

As the first-passage time depends on the particular value of the dissociation rate of the protein-DNA complex, this minimum search time varies parametrically with it, too. Figure 7 illustrates the mean first-passage time to arrive at the 50*^th^* base (left axis) as a function of the dissociation/reaction rate for one particular realization of the binding free energy landscape (shown within the inset of Fig. 3). On the other axis (right axis), estimates for the minimum overall search time are provided, calculated according to equation 17, with the maximum hopping frequency given by *ν* = 10^8^ Hz.

Following the exposition in [16], the search time can be estimated using in vitro experimental protein-DNA binding rates in water, from which the binding rate in cytoplasm can be estimated as *k_on_* ≈ 10^8^ – 10^9^*M*^–1^*s*^–1^. Assuming a cytoplasm transcription factor concentration of ≈ 10^–9^*M*, an order-of-magnitude estimate can be made for the time needed for a protein to bind one site [16]:

This value is in good agreement with the values predicted by the diffusion-reaction model presented here, as shown by the right axis of Fig. 7. Specifically, to agree with the lower bound of such estimates (i.e. ~ 1 second), the reaction rate should be bounded from above by *σ* ≤ 10^–3^, placing it near the plateau region of figure 7.

There are two power-law type regimes here, *d* = 0 and *d* = –0.53, for scaling of type *τ*_50_ ~ *σ^d^*. Here, the exponent of *d* ≈ -1*/*2 means that a 100-fold increase in the reaction rate reduces the search time by 10-fold in the regime characterized by *σ* > 10^–4^. Moreover, the transition the two regimes is abrupt, occuring across a decade or so in the reaction rate. The intersection of these power laws occurs at *σ* ≈ 10^–4^, indicating that a significant number of molecules survive along the segment in biological conditions (refer to the half-life estimates of Fig. 6), so that dissociations of the protein-DNA complex during the search along nonspecific DNA are infrequent.

These two regimes can be interpreted as screening regimes, in that proteins are prevented from reaching downstream sites due to dissociation of the protein-DNA complex. The abrupt transition between these regimes can be attributed to a partial screening regime, wherein exploration is limited by the reaction rate, and representing a balance between maximizing the speed of the sliding phase while allowing for the greatest chance to arrive downstream. Biological advantage would appear to rest somewhere near this transition.

The plateau in the search times for *σ* < 10^–4^, suggests that it may be experimentally difficult to measure reaction rates for these semi-stable comlexes, because the first-passage times vary very little across several orders of magnitude studied. In other words, the relationship between search times and reaction rate is not invertible in the region defined by *σ* < 10^–4^. Nevertheless, the search time associated with the plateau, approximately *t_min_* ≈ 2.4 seconds, sits comfortably within the bound of the experimental estimate provided above.

### Validity of the first-passage renormalization approach

**Figure 8:**
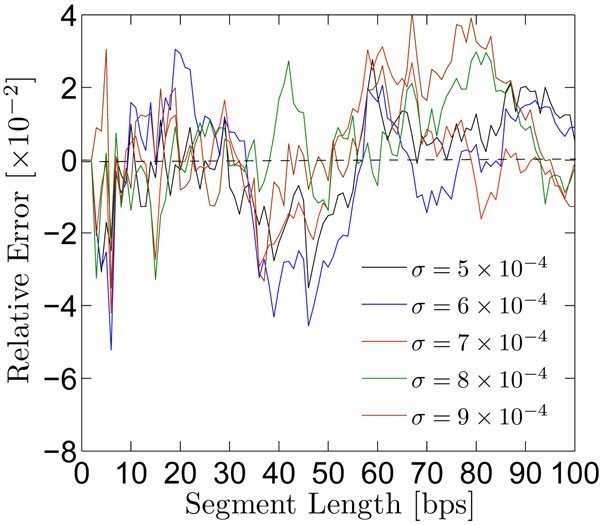
**Validity of the difference-differential transport equations**. Relative errors between mean first-passage times to each site along the model DNA segment, as computed by exact Monte Carlo simulations of the diffusive motion and the difference-differential transport equations.

Exact simulations of the diffusion-reaction process were carried out using MCRW, and the mean first-passage times compared to those given by the renormalization approach, equation 15. These simulations assess the validity of the renormalization approach to the first-passage distribution, because the evaluation of the diffusive process presented in this article is carried out in two independent ways: through the use of difference-differential equations, and through direct simulation using MCRW. Figure 8 illustrates the relative error between these independent methods, wherein they agree to good numerical approximation (to within 5%). The advantage of the difference-differential equation approach to the diffusion-reaction problem considered here, is that it allows for the timely estimates of many diffusional characteristics in the reactive regions that are unaccessible by MCRW due to excessively long simulation run-times.

## Conclusions

A one-dimensional diffusion-reaction model, in the form of a set of difference-differential equations has been proposed to analyze the nonequilibrium protein sliding kinetics along a segment of bacterial DNA. An iterative renormalization procedure was used to express the mean first-passage time to arrive at a downstream site in terms of the occupation probabilities provided by the transport equations. The validity of this approach was established by implementing a Monte Carlo type algorithm that directly compared the renormalization approach to first-passage times measured by the exact simulations.

We found that downstream targets are screened from access by upstream proteins according to the value of the dissociation rate of the protein-DNA complex; however, anomalous characteristics of the surviving associated proteins allow for faster search times as compared to their reaction-free counterparts. Moreover, access limitations induced by dissociation of the protein-DNA complex suggests that nature balances downstream exploration with the competing need for faster search times, possibly allowing for the most efficient use of resources, i.e., the few transcription factors typically present for each gene.

Indeed, experimental estimates of overall target-location times are in good agreement with a minimum bound presented here, indicating that the entire search process is flexible enough to handle a certain amount of variability associated with the increased rarity of DNA-bound proteins (depending on the actual reaction rate). It is clear from these analyses that sliding alone cannot univocally minimize the overall search times, and that other transport mechanisms, such as micro- and macro-hopping events and intersegment transfers, must assist in the search process to provide reliable delivery of proteins to target sites–a result that is consistent with current experimental and theoretical data. Further work is needed, however, to reveal the exact proportion in which these separate mechanisms contribute to the overall search process.

## Competing interests

The authors declare that they have no competing interests.

## Authors' contributions

MLM, EJP, and PG conceived the study; MLM and PG designed and conducted research; MLM, EJP, and PG wrote the paper.
